# *Candida auris*-associated hospitalizations and outbreaks, China, 2018–2023

**DOI:** 10.1080/22221751.2024.2302843

**Published:** 2024-01-18

**Authors:** Jian Bing, Han Du, Penghao Guo, Tianren Hu, Meng Xiao, Sha Lu, Clarissa J. Nobile, Haiqing Chu, Guanghua Huang

**Affiliations:** a Department of Respiratory and Critical Care Medicine, Shanghai Pulmonary Hospital, School of Medicine, Tongji University, Shanghai, People’s Republic of China; b Department of Infectious Diseases, Huashan Hospital, Shanghai Institute of Infectious Disease and Biosecurity, State Key Laboratory of Genetic Engineering, School of Life Sciences, Fudan University, Shanghai, People’s Republic of China; c Department of Clinical Laboratory, The First Affiliated Hospital, Sun Yat-sen University, Guangzhou, People’s Republic of China; d Department of Laboratory Medicine, Sate Key Laboratory of Complex Severe and Rare Diseases, Peking Union Medical College Hospital, Chinese Academy of Medical Sciences, Beijing, People’s Republic of China; e Department of Dermatology and Venereology, Sun Yat-sen Memorial Hospital of Sun Yat-sen University, Guangzhou, People’s Republic of China; f Department of Molecular and Cell Biology, University of California, Merced, Merced, USA; g Health Sciences Research Institute, University of California, Merced, Merced, USA; h Shanghai Key Laboratory of Tuberculosis, Shanghai Pulmonary Hospital, School of Medicine, Tongji University, Shanghai, People’s Republic of China

**Keywords:** *Candida auris*, emerging fungal pathogen, China, outbreaks, molecular epidemiology

## Abstract

The emerging human fungal pathogen *Candida auris* has become a serious threat to public health. This pathogen has spread to 10 provinces in China as of December 2023. Here we describe 312 *C. auris*-associated hospitalizations and 4 outbreaks in healthcare settings in China from 2018 to 2023. Three genetic clades of *C. auris* have been identified during this period. Molecular epidemiological analyses indicate that *C. auris* has been introduced and local transmission has occurred in multiple instances in China. Most *C. auris* isolated from China (98.7%) exhibited resistance to fluconazole, while only a small subset of strains were resistant to amphotericin B (4.2%) and caspofungin (2.2%).

## Dear editor

The emerging multidrug-resistant fungal pathogen *Candida auris* is becoming a serious threat to public health worldwide. Since its first description as a pathogen in Japan in 2009 [1], *C. auris* infection cases have been detected in at least 50 countries across six continents to date [2,3]. Due to its multidrug-resistant properties, difficulties in diagnosing and treating its infections, and high transmission rates in healthcare facilities, *C. auris* has been deemed an “urgent threat” by the CDC and placed on the “critical priority” fungal pathogens list by the World Health Organization (WHO) in 2022. The first detection of *C. auris* in China was reported in 2018, which involved 3 hospitals and 18 cases [4-6].

**Figure 1.**
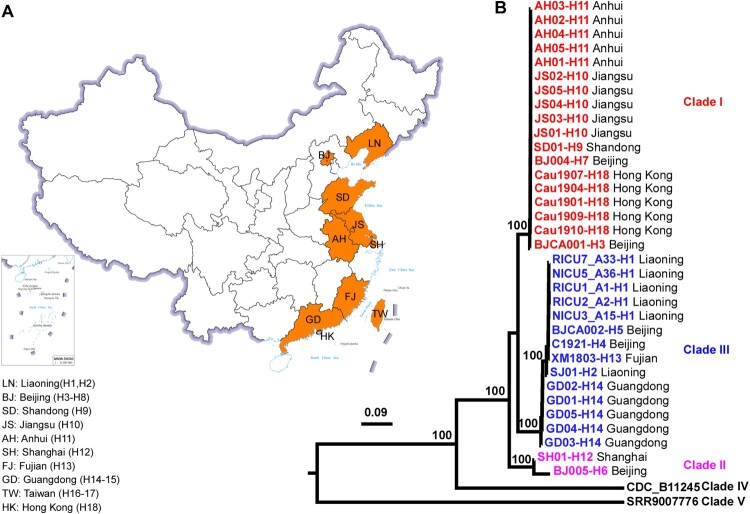
Maximum-likelihood phylogeny analysis of representative *C. auris* strains isolated from different hospitals in China based on genomic sequences. Phylogenetic tree of 34 representative *C. auris* strains from China were constructed based on whole genome SNPs and 1000 bootstrap replicates. Data of *C. auris* clade IV and clade V strains were retrieved from the NCBI SRA database. The hospitals (H1–H18) and provinces from which the *C. auris* strains were isolated are also shown. Strains of clade I, II, and III isolated in China are indicated in red, pink, and blue, respectively.

Here we describe the *C. auris* cases collected in 18 hospitals in China from 2018 to 2023 and present the genomic, epidemiological, clinical, and biological features of the *C. auris* strains associated with these cases. A national retrospective survey on *C. auris* infections in China from January 2016 to August 2023 was performed and a total of 312 cases were collected from 18 hospitals in 10 provinces (Figures 1A and S1). Of these reported cases, 224 (71.8%) were symptomatic cases and 88 (28.2%) were obtained through screening measures. While relatively few cases were identified in 2020 and 2021 (possibly due to enhanced disinfection measures adopted in hospitals for COVID-19 prevention and decreased international travels during this period in China), a rapid rise in cases was observed in 2023 (Figure S1A). Of the 312 cases collected, 70 were reported previously [4–8] and thus we included the existing clinical information and information on the associated *C. auris* isolates in our genomic and phylogenetic analyses. Most cases (280, 89.7%) were associated with four outbreaks in four different hospitals (H1 in Liaoning, H10 in Jiangsu, H11 in Anhui, and H14 in Guangdong). Based on the analysis of the H14 outbreak, 75.0% (105/140) of cases involved patients in the intensive care unit (ICU). Of the 140 *C. auris* positive cases in the H14 outbreak, the age range of affected patients was 3–91 years of age (median age = 65), with 47.9% of patients (67/140) being ≥ 65 years of age. Major risk factors in these patients included lung infections, hypertension, liver diseases, diabetes mellitus, and cancer.

All *C. auris* isolates were subjected to molecular identification by sequencing the ITS and D1/D2 genomic regions. A subset of strains (30) were selected for whole genome sequencing. In total, 53 genomic sequences of clinical strains (23 previously sequenced and 30 sequenced in this study, Dataset S1) representing 51 independent cases from China in addition to 2 environmental strains were analyzed. Based on sequencing results from the ITS and D1/D2 regions and the whole genome, we identified three genetic clades of *C. auris* (I, II, and III; Figure 1B). Isolates of the outbreak cases in hospitals H1 and H14 belonged to clade III, whereas those of the outbreak cases in hospitals H10 and H11 belonged to clade I. Isolates belonging to clades I, II, and III were identified in the other sporadic cases involving all provinces (Figure S1B).

Molecular epidemiological analyses indicated that the clade I isolates of the H10 and H11 hospital outbreaks were closely associated and genetically related to South African and Kenyan strains (Figure S2). Only 23–45 single nucleotide polymorphism (SNP) differences were identified among the strains from these two hospitals (Figure S3), even though the two hospitals are located in different provinces. The clade III isolates of the H1 and H14 hospital outbreaks belonged to two distinct clusters (Figure S4) with 66–84 SNP differences (Figure S3B), suggesting that these two outbreaks had different origins. We note that the first reported case of the H14 hospital outbreak was a travel-related case. This patient had been admitted to a hospital in South Africa, where clade III strains are prevalent [9], and likely acquired *C. auris* from this exposure. Moreover, the *C. auris* strains isolated from environmental surfaces of the H1 and H11 hospitals showed close genetic relatedness to the corresponding clinical isolates from the same hospitals, supporting the idea that these outbreak strains are of clonal origin and that hospital transmission occurred.

Interestingly, although the 15 screening cases caused by clade I strains (isolated from the skin; some were from outpatients) [8], were identified in the same H18 hospital in Hong Kong, the associated strains exhibited high genetic diversity (112–401 SNP differences) (Figure S3A). This finding indicates that these strains are not genetically associated and likely have different origins.

For the sporadic cases, all three clade II strains were isolated from the ear canals of outpatients from H6, H12, and H14 hospitals (the associated patients had ear infection (otitis) but were not admitted to the hospitals). This finding is consistent with previous publications, which demonstrated that clade II strains are associated with the ear niche [1,10,11], suggesting that clade II strains could be ear commensals. The isolate from the H13 hospital in Fujian was closely related to the outbreak strains from the H1 hospital in Liaoning. A comparison of SNP differences among paired isolates of the same genetic clade from the same hospital or two different hospitals is presented in Figure S3B. Strain BJCA001 isolated from the H3 hospital in Beijing [6] was highly divergent from the other clade I strains (>1000 SNPs, Figure S3C). BJCA001 is genetically related to an isolate from the United States (Accession # SRR7909420) and its genome contains over 60 Zorro-3 retrotransposons, which could cause genome instability. The genetic diversity among clade I strains was clearly higher than that of clade III strains. A possible explanation for this finding is that clade I strains often carry more Zorro-3 retrotransposons than clade III strains [12]. Taken together, our findings indicate that there were multiple origins or introductions of *C. auris* into China.

Antifungal susceptibility analyses demonstrated that most of the *C. auris* isolates (308/312, 98.7%) were resistant to fluconazole according to the tentative CDC MIC breakpoints (https://www.cdc.gov/fungal/candida-auris). Only four isolates (4/312, 1.3%, three of clade I and one of clade II) were susceptible to fluconazole. All isolates tested showed a low MIC to 5-fluorocytosine (<0.25 µg/mL). Of all isolates from China, 13 isolates (13/312, 4.2%) were resistant to amphotericin B and seven (7/312, 2.2%) were resistant to caspofungin.

We next analyzed the mutations of antifungal resistance-associated genes. The VF125AL hotspot mutation of *ERG11* (encoding the azole target enzyme 14-alpha-demethylase) was found in all clade III isolates, and the Y132F hotspot mutation of *ERG11* was found in all clade I strains resistant to fluconazole. For the two fluconazole-resistant strains of clade II (H6 and H12), no hotspot mutations were found in *ERG11*; however, we found a mutation (M653I) in *TAC1B* in these strains. This gene has been previously reported to be associated with fluconazole resistance in *C. auris* [13]. The association of the M653I mutation in *TAC1B* and its resistance mechanism remains to be investigated. The caspofungin-resistant isolates carried the S639F hotspot mutation of *FKS1* (encoding the echinocandin target enzyme β-1,3-glucan synthase).

## Conclusion

Despite the rapid rise of *C. auris* cases in China, the incidence of *C. auris* infections is still fewer than those reported in the United States, South Africa, and India. Most cases in China were identified in the east in provinces with comparatively developed economies, where hospital clinical microbiology laboratories have advanced instruments and skilled staff. Given the known difficulties in accurately diagnosing *C. auris* infections, these infections could be significantly underestimated in healthcare settings throughout China.

This study represents the first molecular epidemiological description of *C. auris* isolates in China from 2018 to 2023. Molecular epidemiological analyses suggest that *C. auris* isolates in China could have multiple origins; however, the exact origins in most hospitals are still unclear. Unlike *C. auris* isolates from the United States and India, most *C. auris* isolates from China are only resistant to fluconazole and are susceptible to amphotericin B and caspofungin. The underlying reasons for this antifungal drug resistance profile remain to be investigated. Due to its increased incidence worldwide and the fact that several outbreaks have occurred recently in healthcare settings, it is critical to increase awareness of the emerging threat of *C. auris* to public health.

## Ethics statement

Ethics approval was not required for the genomic and biological analyses of *C. auris* isolates. Deidentified health records were analyzed with approval from the hospital ethics committee.

## Supplementary Material

04_DataSetS1_20231226

Supporting_materials_EMI_2302843
