# Supplementary material for: Candida auris-associated hospitalizations and outbreaks, China, 2018–2023
Source: Emerg Microbes Infect. 2024 Jan 18;13(1):2302843. doi: 10.1080/22221751.2024.2302843 (PMC10802803; doi:10.1080/22221751.2024.2302843)
Supplement: Supporting_materials_EMI_2302843 [file TEMI_A_2302843_SM6776.pdf]

Supporting information for

Candida auris-associated hospitalizations and outbreaks, China, 2018-2023

Jian Bing[1,2#], Han Du[2,#], Penghao Guo[3,#], Tianren Hu[2,#], , Meng Xiao[4], Sha Lu[5], Clarissa J. Nobile[6,7], Haiqing Chu[1,8*], and Guanghua Huang[2,*]

Table S1. Percentage of resistant strains of *C. auris* isolated from China.

Drug	Fluconazole	Amphotericin B	Caspofungin
Number (percentage)	308 (98.7%)	13 (4.2%)	7 (2.2%)

* In total, 312 isolates were analyzed. The CDC tentative MIC breakpoints were used. The fluconazole-susceptible strains were from hospitals H3 (clade I), H14 (clade II), H16 (Clade I), and H17 (Clade I). The amphotericin B-resistant strains were from hospitals H5 (clade III), H9 (clade I), H10 (clade I), and H11 (clade I). The caspofungin-resistant strains were from H1 (clade III), H8 (clade I), H10 (clade I) and H14 (clade III).

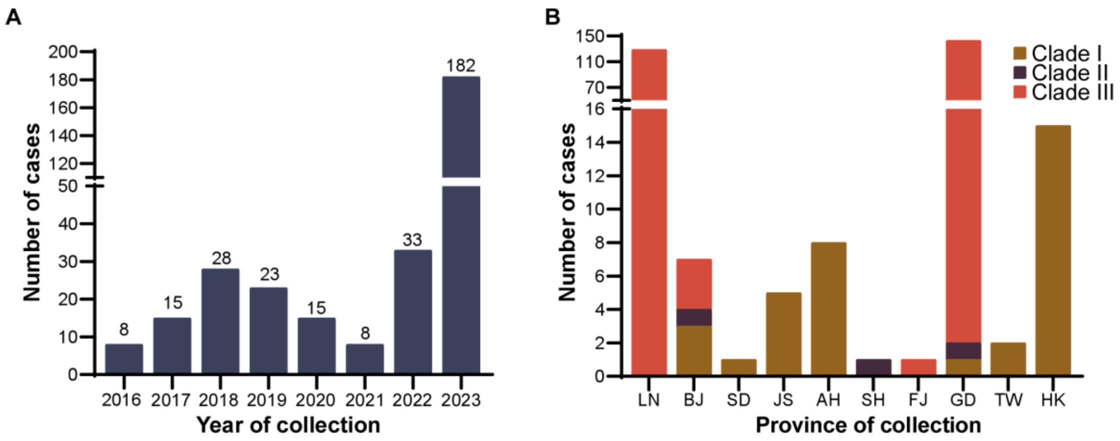

Figure S1. ***Candida auris*** **cases reported in China from 2016-2023.** (A) Number of *C. auris* cases each year. (B) Number of *C. auris* cases identified in the ten provinces. Provinces: LN, Liaoning; BJ, Beijing; SD, Shandong; JS, Jiangsu; AH, Anhui; SH, Shanghai; FJ, Fujian; GD, Guangdong; TW, Taiwan； HK, Hong Kong.

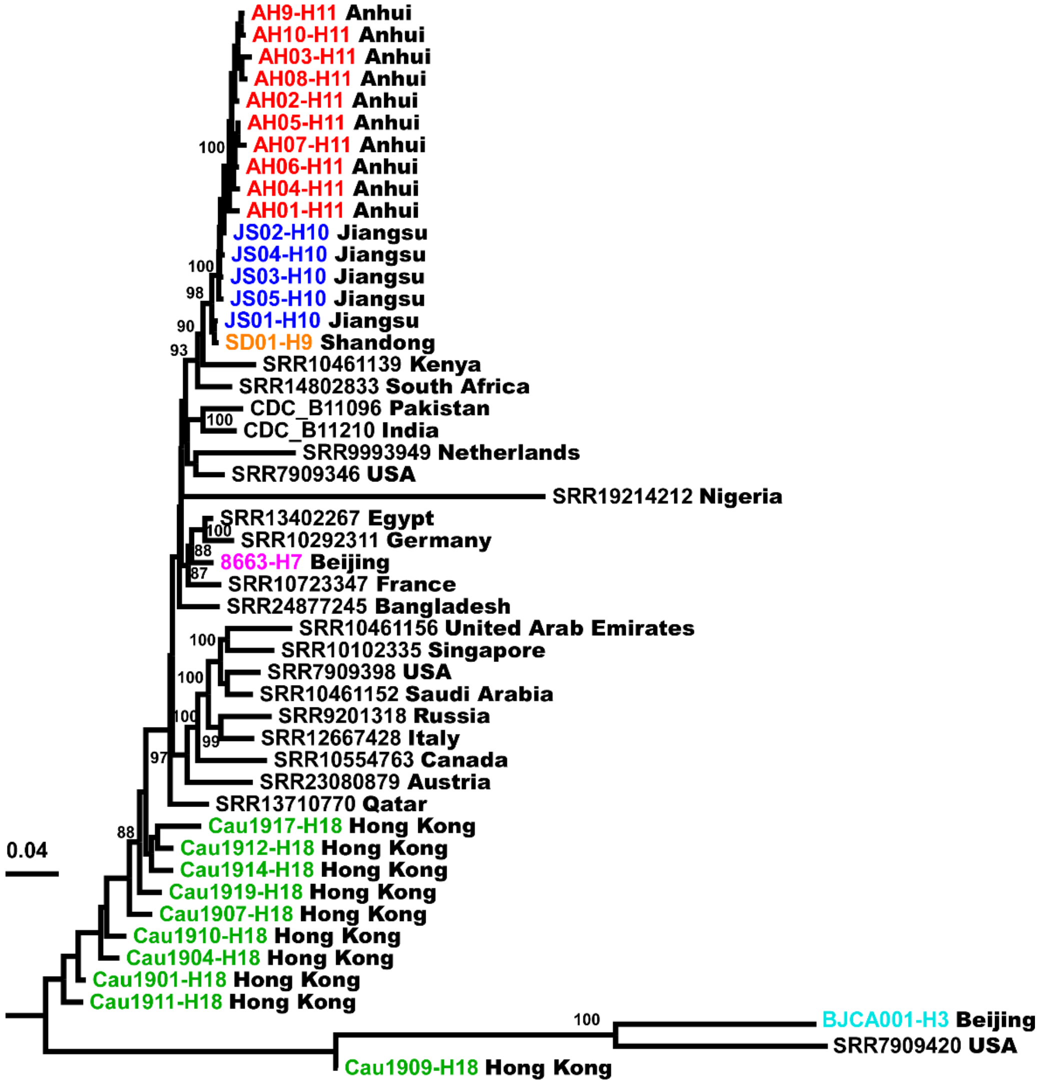

Figure S2. Phylogenetic analysis of *C. auris* clade I strains isolated in China and representative strains from other countries. Maximum-likelihood phylogeny tree was constructed based on whole genome SNPs and 1,000 bootstrap replicates. The genomic data of *C. auris* strains from other countries were retrieved from the NCBI SRA database. GCA_002759435.2 (CDC_B8441, clade I) served as a reference for genome alignment analysis.

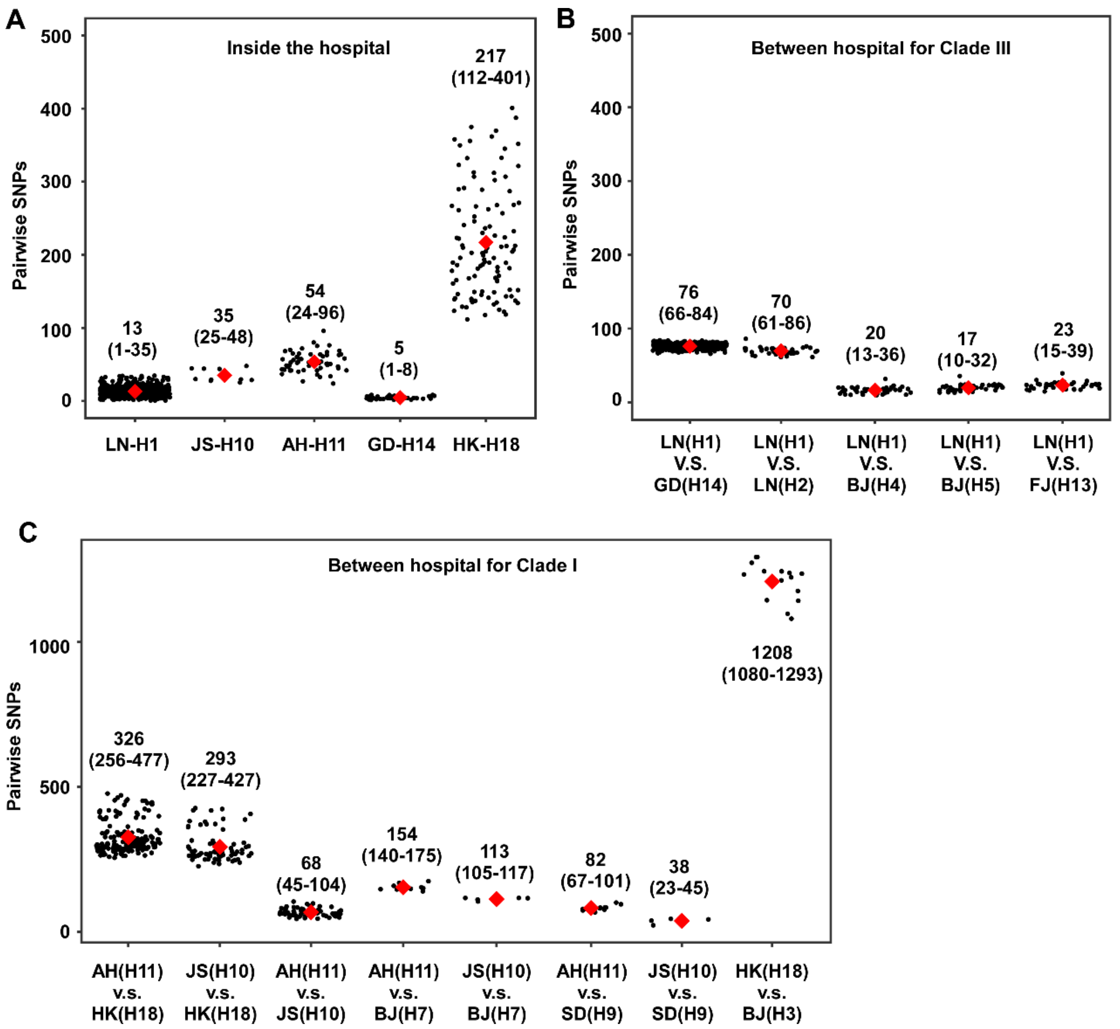

Figure S3. SNP differences among *C. auris* strains of the same clades. (A) The SNP differences among strains of the same clade isolated from the same hospital. AH, Anhui; BJ, Beijing; GD, Guangdong; HK, Hong Kong; JS, Jiangsu; LN, Liaoning (H1); SD, Shandong. (B and C) Pairwise comparison of the SNP differences among strains of clade I (panel B) or clade III (panel C) isolated from two different hospitals. SNPs were based on whole genome analysis. To avoid alignment errors, the genomic data of clade I and III were aligned to GCA_002759435.2 (CDC_B8441, clade I) and GCA_002775015.1 (CDC_B11221, clade III), respectively. The average and range of pairwise SNPs are shown in red.

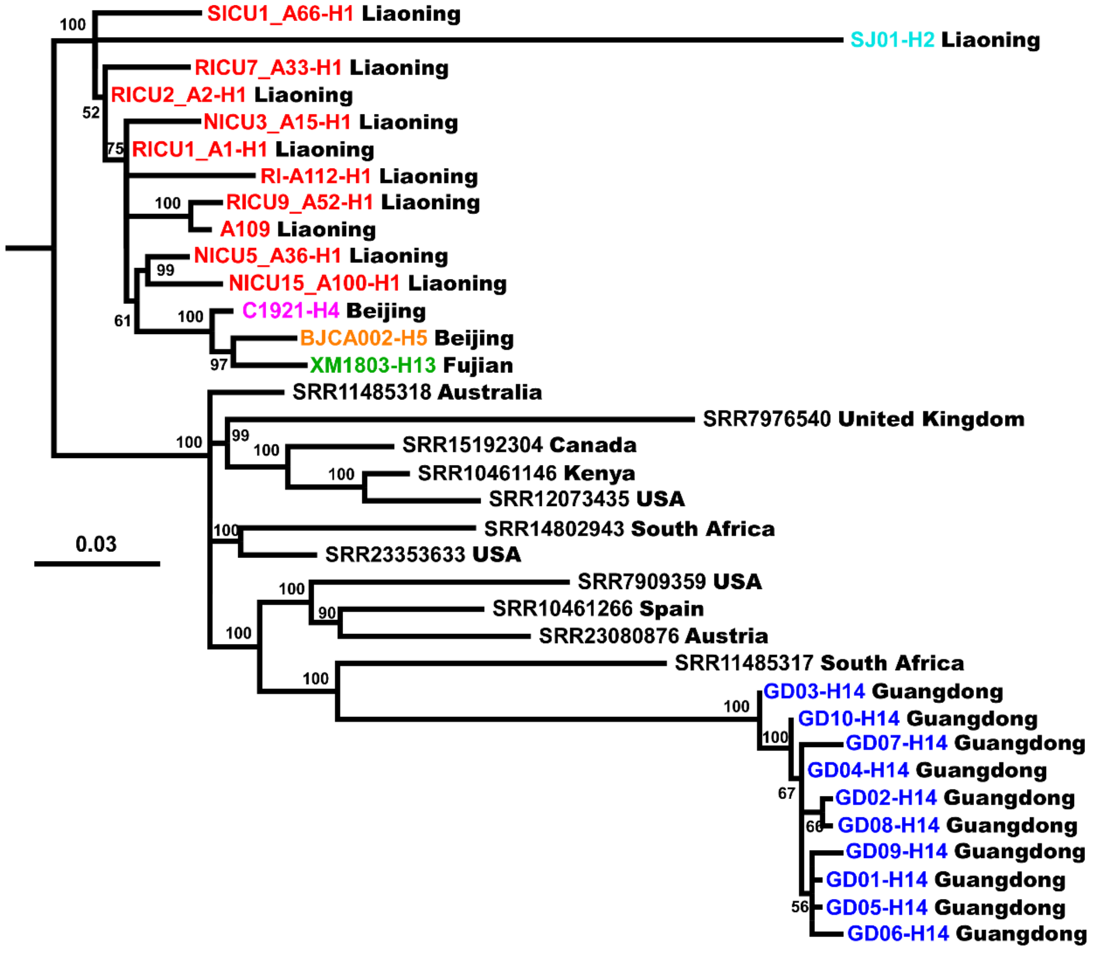

Figure S4. Phylogenetic analysis of *C. auris* clade III strains isolated in China. Maximum-likelihood phylogeny tree was constructed based on whole genome SNPs and 1,000 bootstrap replicates. The genomic data of *C. auris* strains from other countries were retrieved from the NCBI SRA database. GCA_002775015.1 (CDC_B11221, clade III) served as a reference for genome alignment analysis.

Methods

Additional Information and Case Details

Symptomatic cases were defined as those with obvious symptoms of infection and where *C. auris* was isolated from the respiratory tracts, urinary tracts, blood, or other deep organs. Screening cases were defined as those with no obvious symptoms of infection and where *C. auris* was isolated from the skin, mouth swabs, or feces.

H11 outbreak: eight *C. auris* cases (six male and two female), main isolation sites (groin and armpit).

H10 cases: five cases, 34-53 years old (median age = 47). Four cases were blood infections and one was pancreatic infection.

H5 case: strain BJCA002, isolated from the blood sample of a male infant.

Strain culturing and antifungal susceptibility testing

Fungal strains were routinely cultured in YPD medium. The antifungal susceptibility assay was performed using a broth microdilution method as described in the Clinical and Laboratory Standards Institute (CLSI) M27 guidelines. Antifungal powders were purchased from Sigma-Aldrich (St. Louis, MO, USA). *Candida krusei* ATCC 6258 and *Candida parapsilosis* ATCC 22019 served as quality controls.

Whole genome sequencing and data analysis

The *C. auris* isolates selected for whole genome sequencing analysis were based the genetic clade, isolation time, and regions/hospitals of origin. Detailed

description of the sequenced *C. auris* isolates are presented in supplementary **Dataset S1**. One representative isolate for each case was used for biological and molecular epidemiological analyses. Genomic DNA of *C. auris* was extracted using the TIANamp Yeast DNA Kit (TianGen Biotech, Beijing, China) according to the manufacturer's protocol. The BGISEQ-500 sequencer was used for sequencing. Genomic analysis was based on our previous publication [1]. Briefly, raw reads were trimmed to remove low-quality (phred score ≤10), ambiguous, and adaptor sequences using the FASTX-Toolkit v0.0.14 (http://hannonlab.cshl.edu/fastx_toolkit/index.html). The clean reads were then mapped to the reference genome GCA_002759435.2 (CDC_B8441, clade I) or GCA_002775015.1 (CDC_B11221, clade III) using BWA mem 0.7.17 software with default settings. SAMTools v1.361, Picard Tools v1.56 (http://picard.source-forge.net), and GATK v2.7.2 were used for SNP and INDEL analyses [2-4] . The SNP difference numbers among different strains were calculated using an in-house script.

Public available genomic data of *C. auris* were retrieved from the NCBI SRA database (https://www.ncbi.nlm.nih.gov/sra, see **Dataset S1** for details).

Phylogenetic analysis

The Maximum-likelihood phylogenetic tree was constructed using RAxML v7.3.227 based on whole genome SNPs with 1,000 bootstrap replicates [5]. The General Time Reversible (GTR) model and Gamma distribution with Invariant sites (G+I) were used. The SRA accession numbers of *C. auris*

genomic data used for analysis are presented in supplementary **Dataset S1**.

Supplementary references

[1] Bing J, Guan Z, Zheng T, et al. Clinical isolates of Candida auris with enhanced adherence and biofilm formation due to genomic amplification of ALS4. PLoS Pathog. 2023 Mar;19(3):e1011239.

[2] Li H, Durbin R. Fast and accurate short read alignment with Burrows-Wheeler transform. Bioinformatics. 2009 Jul 15;25(14):1754-60.

[3] Li H, Handsaker B, Wysoker A, et al. The Sequence Alignment/Map format and SAMtools. Bioinformatics. 2009 Aug 15;25(16):2078-9.

[4] McKenna A, Hanna M, Banks E, et al. The Genome Analysis Toolkit: a MapReduce framework for analyzing next-generation DNA sequencing data. Genome research. 2010 Sep;20(9):1297-303.

[5] Stamatakis A. RAxML-VI-HPC: maximum likelihood-based phylogenetic analyses with thousands of taxa and mixed models. Bioinformatics. 2006 Nov 1;22(21):2688-90.
